# Association between CEBPE Variant and Childhood Acute Leukemia Risk: Evidence from a Meta-Analysis of 22 Studies

**DOI:** 10.1371/journal.pone.0125657

**Published:** 2015-05-04

**Authors:** Jian Sun, Jinyu Zheng, Linjun Tang, Jasmine Healy, Daniel Sinnett, Yue-e Dai

**Affiliations:** 1 Department of Anesthesiology, Huai’an Matenal and Child Health Hospital, Huai’an, Jiangsu, China; 2 Department of Neurosurgery, The Affiliated Huai'an Hospital of Xuzhou Medical College, Huai'an, China; 3 Department of Neurosurgery, Tongling People's Hospital, Tongling, An’hui, People's Republic of China; 4 Sainte-Justine University Hospital Research Center, Montreal, Quebec, Canada; 5 Department of Pediatrics, Faculty of Medicine, University of Montreal, Montreal, Quebec, Canada; 6 Nanjing Children’s Hospital, Affiliated with Nanjing Medical University, Nanjing, Jiangsu, People’s Republic of China; Queen's University Belfast, UNITED KINGDOM

## Abstract

The CCAAT/enhancer binding proteins (CEBPs) have been involved in the etiology of acute leukemia (AL) and investigated in numerous genetic association studies, however, the results were inconclusive. The current meta-analysis was conducted to clarify the effect of CEBPE rs2239633 variant on childhood AL risk. Electronic literature search was performed on August 15, 2014, from databases of Medline, PubMed, Embase, and Web of Science. A total of 22 case-control studies were eligible for the pooled analysis. The results demonstrated that rs2239633 A allele was significantly associated with a decreased risk of childhood AL (A vs G: OR=0.87, 95%CI = 0.80, 0.94, p<0.001), especially in B-cell ALL subgroup (A vs G: OR = 0.79, 95%CI = 0.74, 0.83, p<0.001), but not among T-cell ALL or AML subgroups. In the stratified analysis by ethnicity, the association was observed in Europeans (A vs G: OR = 0.80, 95%CI = 0.76, 0.84, p<0.001) but not in Asian and mixed populations. Moreover, the results of sensitivity and cumulative meta-analysis indicated the robustness of our results. Also, Begg’s and Egger’s tests did not indicate any evidence of obvious asymmetry. In summary, our study provided evidence that CEBPE rs2239633 variant is associated with decreased risk of childhood B-cell ALL in Europeans.

## Introduction

Acute leukemia (AL), characterized by dysregulated clonal expansion of immature lymphoid or myeloid progenitor cells, is the most common cancer in children.[1] In the United States, it accounts for~20 000 cancer diagnoses and 10 000 deaths.AL can be subdivided into acute myeloid leukemia (AML) and acute lymphocytic leukemia (ALL) according to the cell type. Studies for leukemogenesis have been conducted for many years, and previous studies provided evidence that infections and immunologic response might play a role in the etiology of childhood leukemia.[2,3] However, the mechanisms underlying the development of most AL remain unclear.[2,4]

The CCAAT/enhancer binding proteins (*CEBPs*) are transcription factors involved in hematopoietic cell development, including granulopoiesis. Akagiet al [5] showed that *CEBPB* and *CEBPE* double-knockout mice were highly susceptible to fatal infections and died within 2–3 months. Also, the proportion of hematopoietic progenitor cells in the bone marrow of the knockout mice was significantly increased. Recently, genome wide association (GWA) studies have identified candidate single nucleotide polymorphism (SNP) located in *CEBPE* (14q11.2), which was strongly related to the susceptibility of childhood ALL.[6,7] Taken together, these results suggested that *CEBPE* might appear to be a good candidate gene for childhood AL. Up to date, an accumulating number of studies focused on the association between *CEBPE* variant and ALL risk, however, the conclusions of these studies were inconsistent. Thus, we conducted a meta-analysis with an overall larger sample size by summarizing previous case-control studies to clarify the associations of polymorphisms in the *CEBPE* gene with susceptibility to childhood AL, including B-cell ALL, T-cell ALL and AML.

## Methods

### Search Strategy

A comprehensive literature search of the Medline, Pubmed, Embase, and Web of Sciencerepositories was carried out using the following keywords: (‘acute leukemia’, ‘ALL’ or ‘acute lymphoblastic leukemia’ ‘acute myeloid leukemia’, ‘AML’), (‘polymorphism’, ‘variant’, ‘mutation’) and (‘*CEBPE*’, or ‘rs2239633’) (the last search update was August 15 2014). Moreover, the reference lists of review articles and retrieved articles were hand-searched for additional potential studies.

### Inclusion and Exclusion Criteria

Studies meeting the following criteria were included in themeta-analysis: (1) reporting the association between *CEBPE* rs2239633 variant and childhood AL risk (2)providing sufficient data to estimate odds ratios (ORs) with 95% confidence intervals (95% CIs). A study was excluded if: (1) had no control population (2) investigated the adult acute leukemia.

### Data Extraction

The following information was gathered for each eligible study by two independent authors: name of the first author, year of publication, number of patients and healthy controls, sex and mean age in patients and healthy controls, country of origin, ethnicity of the individuals involved, method of genotyping, types of acute leukemia (e.g., B-cell ALL, T-cell ALL or AML), allele frequency and genotype frequency. The corresponding author would be contacted when the article did not provide sufficient genotype distributions. Moreover, disagreements were resolved by discussion between the two investigators.

### Statistical Analysis

Odds ratios (ORs) and corresponding 95% confidence intervals (CIs) were estimated to assess the association between *CEBPE* rs2239633 polymorphism and risk of childhood acute leukemia. The significance of the pooled OR was determined by the Z-test, and a P value of less than 0.05 was considered significant. In the control group, the Hardy-Weinberg equilibrium (HWE) was assessed, and a P<0.05 was considered as significant disequilibrium. In addition, subgroup analysis was carried out stratified by types of AL and ethnicity. Between-study heterogeneity was evaluated using the χ^2^-based Q test and I^2^ test.[8,9] A P<0.10 or I^2^>50% was considered significant heterogeneity, and a random effects model (DerSimonian and Laird method) was used, otherwise a fixed effects model was applied.[10] Subgroup analysis, meta-regression analysis and Galbraith plot were carried out to assess the potential source of heterogeneity.[11,12]

Sensitivity analysis by omitting one study at a time was also performed to assess the influence of individual studies on the combined risk estimate.[13] We also carried out a cumulative meta-analysis to measure the genetic effect changes as evidence accumulating over time and measured the trend in estimated risk effect. Potential publication bias was assessed by visual inspection of the Begg’s funnel plot and statistically via Egger’s regression tests.[14,15] If publication bias was detected, we adjusted for the effect by means of Duval and Tweedie’s nonparametric trim-and-fill method.[16] All statistical analyses were performed using the STATA software, version12 (StataCorp LP, College Station, Texas).

## Results

### Main Characteristics of the Included Studies

**Fig 1:**
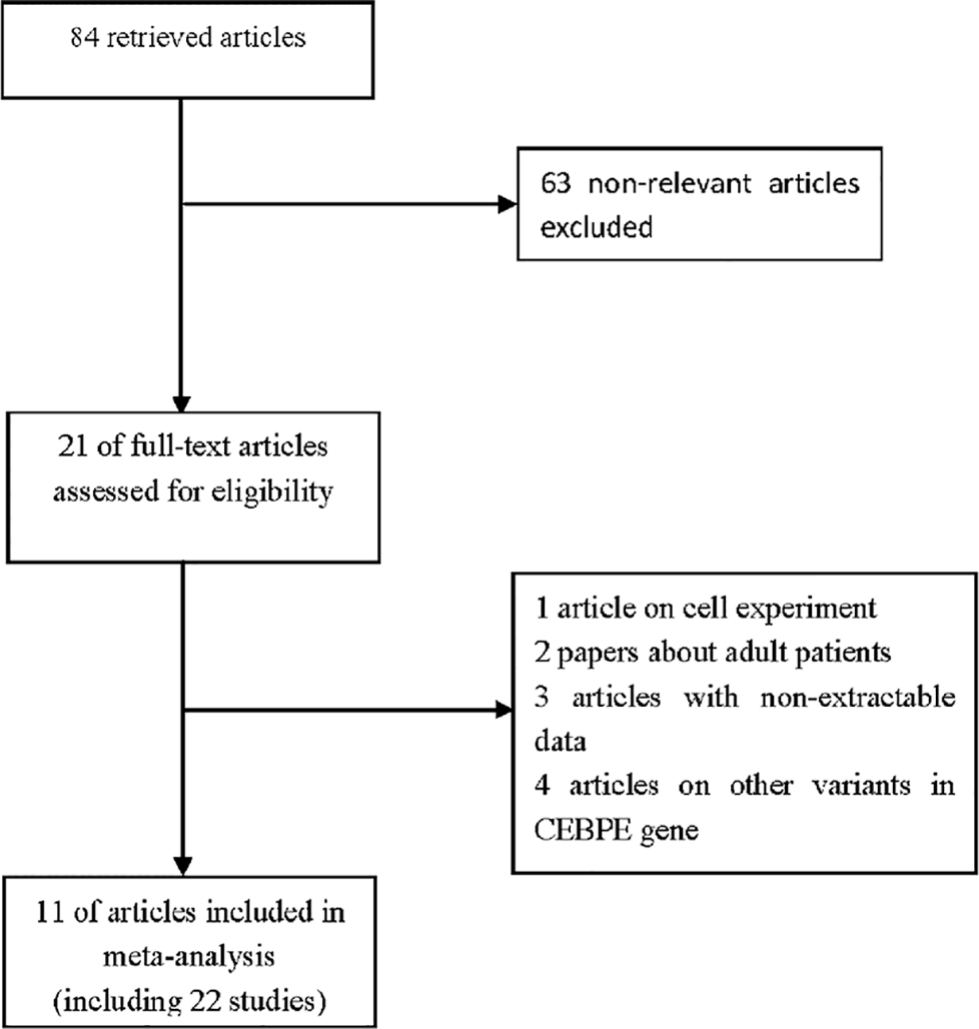
Study selection procedures for a meta-analysis of CEBPE rs2239633 polymorphism and childhood AL risk. AL: acute leukemia.

Eighty-four references were identified during our premature searches, of which, 63 non-relevant articles were excluded following review of title and abstract. In the remaining 21 full text papers, 10 studies did not investigate the association between *CEBPE* rs2239633 polymorphism and childhood AL risk. The selection process is illustrated in Fig 1. Of these eligible articles, 3 publications reported data on different subpopulations,[17–19] 4 investigated on multiple disease types of childhood AL and we treated these studies independently.[7,17,20–22] Thus, a total of 22 studies involving 6152 patients and 11739 healthy controls met our selection criteria. (Table 1)

**Table 1:** Characteristics of studies included in the meta-analysis.

First author	year	country	Ethnicity	Disease	Genotyping Methods	Cases/Controls
Papaemmanuil (GWAS-1)	2009	UK	Europeans	B-cell ALL	Illumina Infinium Human 370Duo BeadChips	459/1438
Papaemmanuil (GWAS-1)	2009	UK	Europeans	T-cell ALL	Illumina Infinium Human 370Duo BeadChips	44/1438
Papaemmanuil (GWAS-2)	2009	UK	Europeans	B-cell ALL	Illumina Infinium Human 370Duo BeadChips	365/960
Papaemmanuil (GWAS-2)	2009	UK	Europeans	T-cell ALL	Illumina Infinium Human 370Duo BeadChips	39/960
Prasad	2010	Germany	Europeans	B-cell ALL	Kaspar allele-specific PCR	1193/1516
Prasad	2010	UK	Europeans	B-cell ALL	Kaspar allele-specific PCR	191/361
Emerenciano	2014	Brasil	Mixed	B-cell ALL	Taqman	77/490
Emerenciano	2014	Brasil	Mixed	B-cell ALL	Taqman	77/490
Emerenciano	2014	Brasil	Mixed	AML	Taqman	93/490
Pastorczak	2011	Poland	Europeans	ALL (88% B-cell)	Taqman	398/731
Ross	2013	USA	Europeans	ALL	Taqman	85/363
Ross	2013	USA	Europeans	AML	Taqman	52/363
Vijayakrishnan	2010	Thailand	Asians	B-cell ALL	Kaspar allele-specific PCR	172/182
Vijayakrishnan	2010	Thailand	Asians	T-cell ALL	Kaspar allele-specific PCR	18/182
Wang	2013	China	Asians	ALL	SNaPshot	570/673
Ellinghaus	2012	Germany	Europeans	B-cell ALL	SNPlex and TaqMan	419/474
Ellinghaus	2012	Germany	Europeans	B-cell ALL	SNPlex and TaqMan	406/1682
Ellinghaus	2012	Italy	Europeans	B-cell ALL	SNPlex and TaqMan	287/579
Healy	2010	Canada	Europeans	B-cell ALL	allele-specific primer extension	284/270
Orsi	2012	France	Europeans	B-cell ALL	Human CNV370-Quad	361/1542
Orsi	2012	France	Europeans	T-cell ALL	Illumina beadchip	41/1542
Lautner-Csorba	2012	Hungary	Europeans	ALL(72% B-cell)	Sequenom iPLEX Gold	543/529
					MassARRAY technology	

ALL: acute lymphoid leukemia; AML: acute myelogenous leukemia.

### Quantitative Synthesis

**Fig 2:**
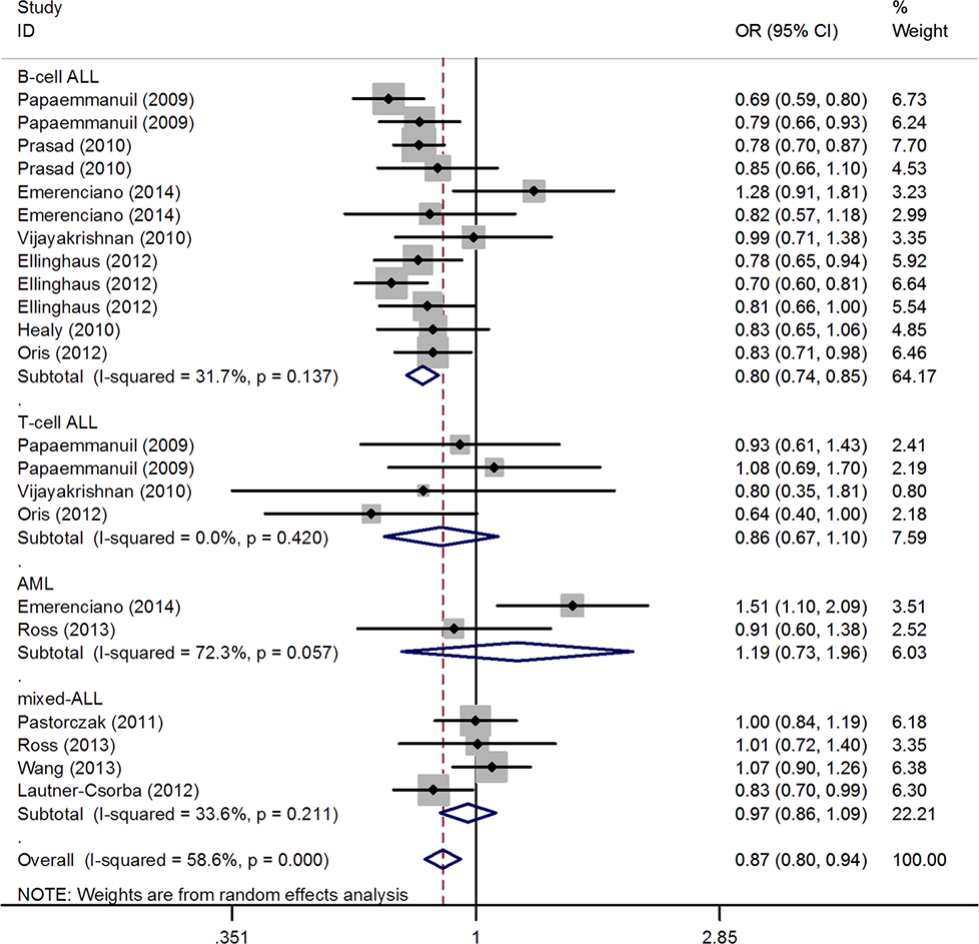
OR estimates with the corresponding 95% CI for the association of CEBPE rs2239633 variant with childhood AL risk (A vs G). The sizes of the squares reflect the weighting of included studies. AL: acute leukemia; OR: odds ratio; CI: confidence interval.

The summary of meta-analysis for the *CEBPE* polymorphism with acute leukemia involving 6152 patients and 11739 healthy controls is shown in Table 2. The results of combined analyses revealed a significant association of rs2239633 variant with acute leukemia risk at the allelic level (A vs G: OR = 0.87, 95%CI = 0.80, 0.94, p<0.001) (Fig 2) and AA vs GG: OR = 0.78, 95%CI = 0.67, 0.93, p = 0.005; at the genotype level under a recessive model (AA vs AG+GG: OR = 0.80, 95%CI = 0.73, 0.88, p<0.001). Moreover, in the stratified analysis by types of acute leukemia and ethnicity, lower risk was detected in the B-cell ALL subgroup (A vs G: OR = 0.79, 95%CI = 0.74, 0.83, p<0.001), ALL subgroup (A vs G: OR = 0.84, 95%CI = 0.79, 0.90, p<0.001) and European subgroup (A vs G: OR = 0.80, 95%CI = 0.76, 0.84, p<0.001). However, in the subgroups of T-cell, AML, Asians, and mixed populations, the results showed no evidence supporting associations between rs2239633 and disease risk. Moreover, a significant association was found in population-based subgroup (A vs G: OR = 0.78, 95%CI = 0.74, 0.83, p<0.001).

**Table 2:** Pooled ORs and 95% CIs for associations between CEBPE rs2239633 polymorphism and childhood AL risk.

**Study group**	A vs G			AA vs GG			AG vs GG			AA+AG vs GG			AA vs AG+GG		
	OR(95%CI)	P_h_	P	OR(95%CI)	P_h_	P	OR(95%CI)	P_h_	P	OR(95%CI)	P_h_	P	OR(95%CI)	P_h_	P
**Total**	0.87(0.80,0.94)	<0.01	<0.01	0.78(0.67,0.93)	<0.01	<0.01	0.91(0.80,1.02)	0.01	0.11	0.88(0.77,1.00)	0.01	0.05	0.80(0.73,0.88)	0.20	<0.01
**Type**															
ALL	0.84(0.79,0.90)	0.01	<0.01	0.74(0.64,0.86)	0.06	<0.01	0.89(0.79,1.00)	0.02	0.05	0.85(0.75,0.96)	<0.01	<0.01	0.78(0.71,0.86)	0.44	
B-cell ALL	0.79(0.74,0.83)	0.14	<0.01	0.64(0.56,0.73)	0.24	<0.01	0.78(0.71,0.86)	0.07	<0.01	0.74(0.68,0.81)	0.04	<0.01	0.75(0.66,0.84)	0.60	<0.01
T-cell ALL	0.86(0.67,1.09)	0.42	0.23	0.68(0.39,1.18)	0.48	0.22	1.06(0.73,1.56)	0.46	0.81	0.95(0.65,1.37)	0.35	0.72	0.65 (0.41,1.05)	0.83	0.08
AML	1.19(0.73,1.96)	0.06	0.48	1.39(0.58,3.36)	0.08	0.46	1.13(0.56,2.29)	0.09	0.72	1.20(0.57,2.50)	0.06	0.63	1.33(0.86,2.06)	0.26	0.20
**Ethnicity**															
Europeans	0.80(0.76,0.84)	0.19	<0.01	0.67(0.60,0.75)	0.25	<0.01	0.81(0.74,0.88)	0.19	<0.01	0.76(0.70,0.83)	0.15	<0.01	0.76(0.69,0.84)	0.46	<0.01
mixed	1.12(0.83,1.67)	0.04	0.36	1.38(0.80,2.39)	0.17	0.25	1.20(0.70,2.07)	0.03	0.51	1.24(0.72,2.12)	0.02	0.44	1.25(0.86,1.81)	0.44	0.25
Asians	1.04(0.90,1.21)	0.74	0.57	1.09(0.78,1.52)	0.82	0.63	1.07(0.87,1.31)	0.62	0.52	1.07(0.88,1.29)	0.67	0.52	1.03(0.75,1.41)	0.76	0.84
**Source of control**															
PB	0.78(0.74,0.83)	0.38	<0.01	0.64(0.56,0.73)	0.48	<0.01	0.78(0.71,0.86)	0.23	<0.01	0.74(0.68,0.81)	0.21	<0.01	0.74(0.66,0.83)	0.80	<0.01
HB	0.97(0.85,1.11)	0.01	0.71	0.98(0.74,1.28)	0.04	0.86	1.02(0.90,1.16)	0.10	0.76	1.00(0.89,1.13)	0.03	0.86	0.93(0.80,1.08)	0.11	0.34

AL: acute leukemia; ALL: acute lymphoid leukemia; AML: acute myelogenous leukemia; CI: confidence interval; OR: Odds ratio; P_h_: P value for heterogeneity.

### Test of Heterogeneity

**Fig 3:**
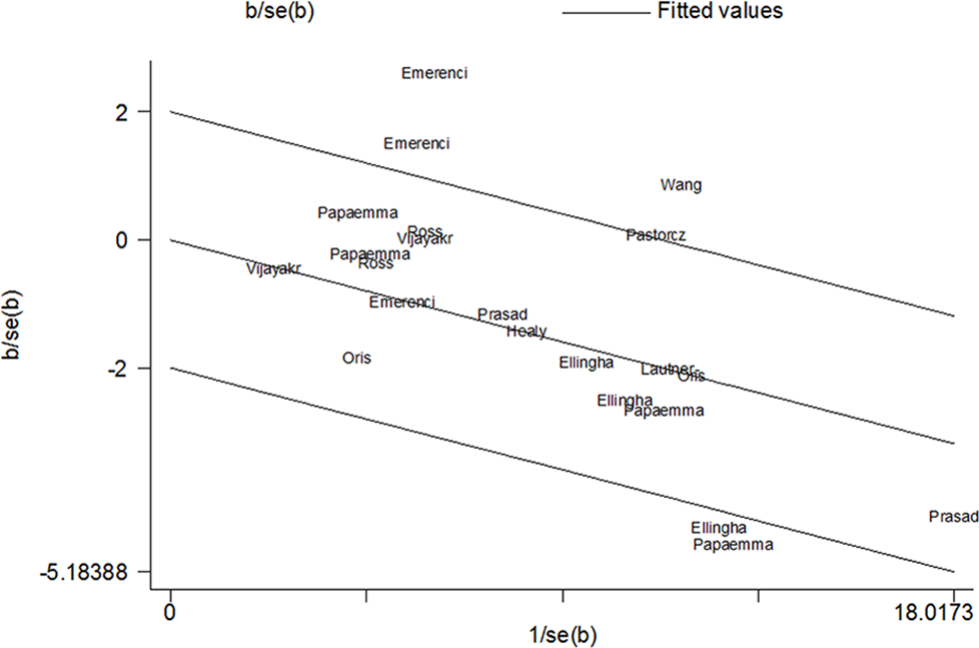
Galbraith plots of CEBPE rs2239633 polymorphism and childhood AL risk. The 95% confidence interval is between the two outer parallel lines at two units above and below the regression line. The outliers were potential sources of heterogeneity.

Significant between-study heterogeneity was detected in all genetic models expect for the recessive model. Further, we performed meta-regression to assess the potential source of heterogeneity by ethnicity, disease types, sample size, and year of publication. The results showed that disease types, source of control, ethnicity and year of publication were the potential sources of heterogeneity, which explained 79% (p = 0.001), 52.8% (p = 0.01), 57.1% (p = 0.006), and 31.2% (p = 0.03) of τ^2^, respectively. Moreover, Galbraith plot analysis indicated studies as outliers, which were possible sources of heterogeneity, when excluded, the heterogeneity was non-significant (Ph = 0.77 for A vs G) and the association was still significant (A vs G: OR = 0.83, 95%CI = 0.79, 0.88, p<0.001). (Fig 3)

### Sensitivity Analyses and Cumulative Meta-Analysis

**Fig 4:**
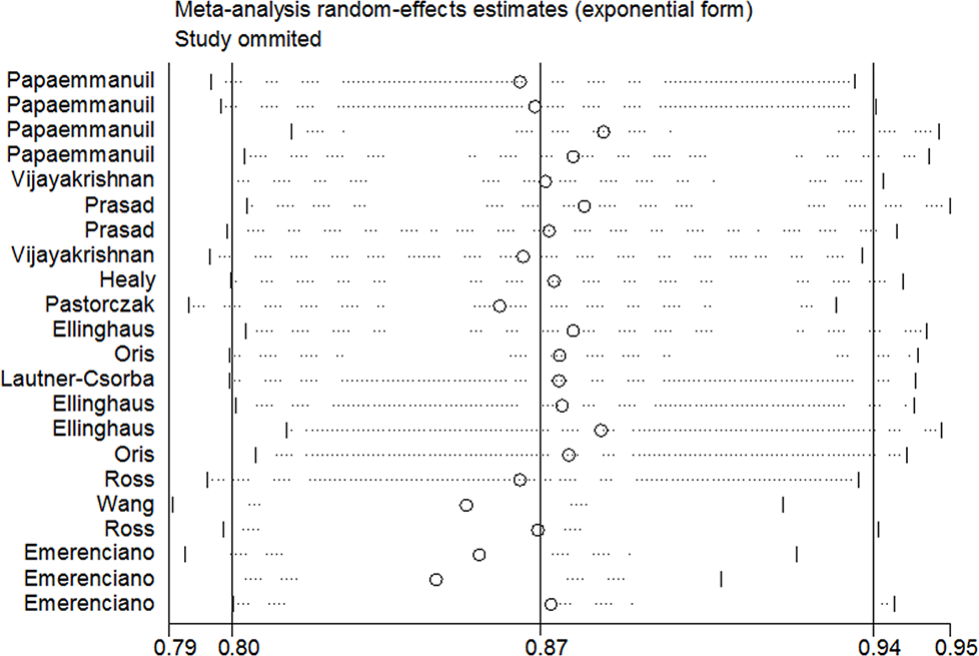
Sensitivity analysis on the association between CEBPE rs2239633 polymorphism and childhood AL risk (A vs G). Results were computed by omitting each study (left column) in turn. Bars: 95% confidence interval. AL: acute leukemia.

**Fig 5:**
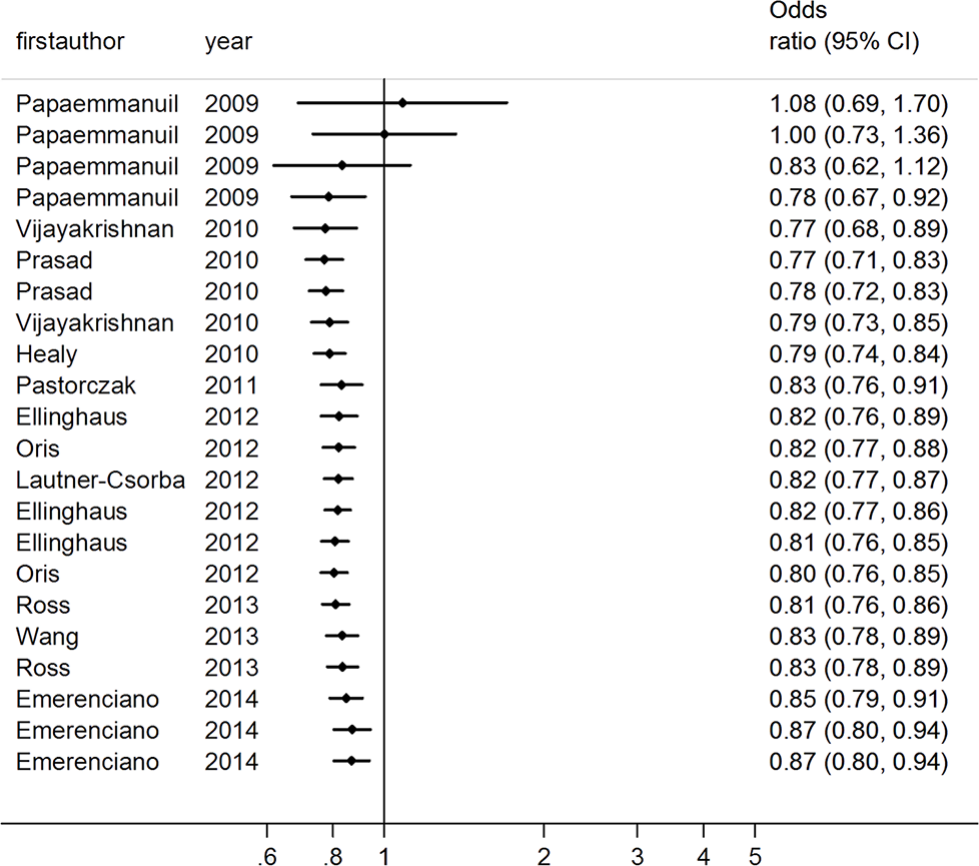
OR estimates with the corresponding 95% CI for the results of cumulative meta-analyses of the association between CEBPE rs2239633 variant and childhood AL risk (A vs G). AL: acute leukemia; OR: odds ratio; CI: confidence interval.

In the sensitivity analysis, the pooled OR did not qualitatively change by omitting a single study at a time. (Fig 4) In addition, the results of cumulative meta-analysis showed that the pooled ORs of rs2239633 variant tended to be stable and the associations tended toward significant associations with accumulation of more data over time, indicating the robustness of our results. (Fig 5)

### Publication Bias

**Fig 6:**
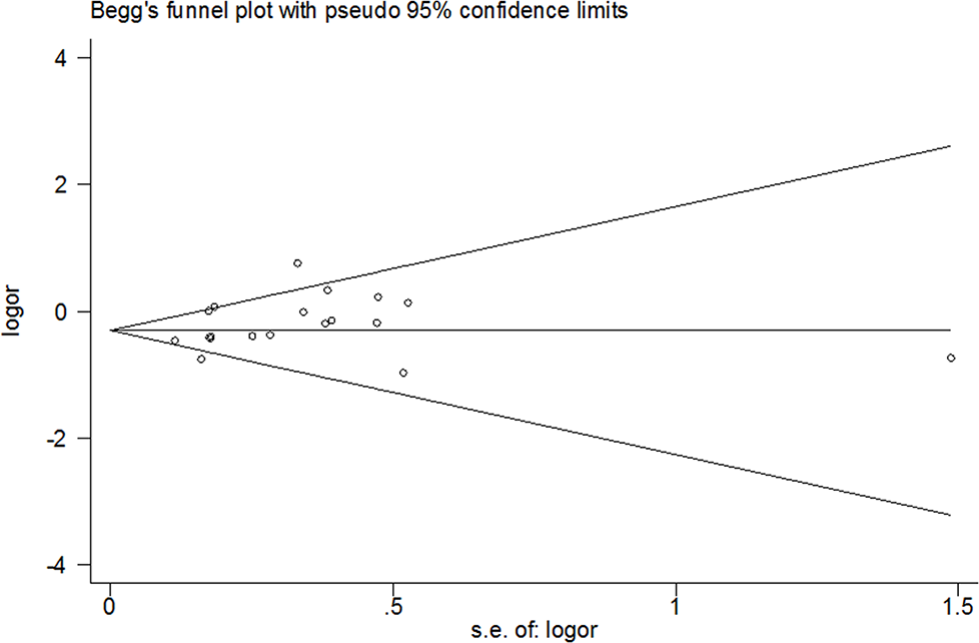
Publication bias in studies of the association between the CEBPE rs2239633 variant and childhood AL risk assessed by Begg’s funnel plot (AA vs GG). AL: acute leukemia; Log(OR): the natural logarithm of the odds ratio.

Publication bias was assessed by Begg’s and Egger’s tests. The shapes of the funnel plots did not indicate any evidence of obvious asymmetry. (Fig 6) Moreover, the results showed no evidence of publication bias in the allelic association test. (AA vs GG: P = 0.18 for Begg’s test, P = 0.12 for Egger’s test)

## Discussion

Recent studies showed that *CEBPE*, along with other 4 *CEBP* family members, have been targeted by recurrent immunoglobulin heavy chain translocations in B-cell precursor ALL, suggesting a possible role of *CEBPE* insusceptibility to ALL.[23] Moreover, the relationship between *CEBPE* rs2239633polymorphism and childhood ALL has been investigated in several genetic association studies, though the results of these studies were contradictory and inconclusive, owing to the small sample sizes and ethnic difference.[17,21,24,25] Meta-analysis is a statistical method, which can overcome the problem of small sample sizes and inadequate statistical power in different studies.[26,27]

In the present meta-analysis, we found a significant association between *CEBPE* variant and childhood ALL risk in the overall population. In the subgroup analysis by ethnicity, the results indicated that *CEBPE* rs2239633 polymorphism was significantly associated with childhood AL in Europeans, but not among mixed and Asian populations, suggesting that the relative contribution of individual susceptibility genes may vary across different populations. Previous studies demonstrated that racial and ethnic disparities persist in the development of ALL.[28] In general, Hispanic whites have the highest incidence of ALL, while blacks are less likely to develop nearly all AL subtypes.[29] Additionally, compared with white and Asian children, black children had the lowest overall survival and event-free survival rates.[30] When the data were stratified by AL subtypes, the significant correlation was only detected in the B-cell ALL subgroup, similar result was observed in a recent meta-analysis. [31] However, the recent meta-analysis did not investigate the association of *CEBPE* variant with susceptibility to T-cell ALL or AML. Here, in the subgroup analysis, significant association was not observed in T-cell ALL (A vs G: OR = 0.86, 95%CI = 0.67, 1.09, p = 0.23) and AML (A vs G: OR = 1.19, 95%CI = 0.73, 1.96, p = 0.48) subgroups. Childhood AL is a group of diseases with varied immunophenotypes and genetic changes. It is suspected that B-cell or T-cell ALL and AML may not share a common etiology.[32] In the US, ALL is diagnosed in approximately 2000 children each year, whereas AML is diagnosed in only about 500 children. Moreover, the cure rate of children with ALL was approximately 90%,[33,34] while the survival in patients with AML was only 60% to 70% in developed countries.[35–37] These findings suggested that *CEBPE* polymorphism was only associated with susceptibility to B-cell ALL subtype, which was also supported by the evidence that *GSTM1* and *XRCC1* Arg399Glnvariants were only associated with ALL but not AML.[38,39] However, in our study, only 4 and 2 studies were eligible for the analysis of T-cell ALL and AML, respectively, which might compromisethe reliability of these findings. Thus, further studies with larger sample size are required to validate the possible role of *CEBPE* polymorphisms in childhood AL, especially in T-cell ALL and AML.

In our meta-analysis, heterogeneity was detected in most genetic models. Thus, we carried out meta-regression and Galbraith plot analyses to assess the sources contributing to the heterogeneity. The results of meta-regression showed that types of disease, source of control, ethnicity and year of publication could explain 79%, 52.8%, 57.1%, and 31.2% of τ^2^, respectively, indicating that these factors were the potential sources of between-study heterogeneity. In addition, the Galbraith plot demonstrated five studies that were the potential origin of heterogeneitysince, when excluded, the heterogeneity was removed. In these studies, disease subtype heterogeneity [17,40] and ethnicity differences [40] might explain some heterogeneity.

This meta-analysis increased statistical power by pooling data from all eligible studies, whereas several limitations should be acknowledged in our meta-analysis. First, we only included studies published in English, which might introduce a language bias. Second, sample size was small for some subgroups, such as T-cell ALL and AML subgroups, which might limit the precision of the pooled estimates, suggesting that more large sample sizes, precise and stratified studies (especially about childhood T-cell ALL and AML are still in urgent need for further evaluation. Moreover, publication bias occurred because we only selected published articles to acquire data for analyses. Also, significant heterogeneity was detected in major comparisons, while disease types and ethnicity were identified as the potential sources of heterogeneity by meta-regression and Galbraith plot analyses. Finally gene-gene and gene–environment interactions were not analyzed due to insufficient data.

Despite these limitations, our results are significant. Our meta-analysis demonstrates that *CEBPE* rs2239633may be associated with susceptibilitytoB-cell ALL in the European population. However, there is lack of evidence showing the correlation between this polymorphism and risk of T-cell ALL and AML, which needs to be validated by further well-designed genetic association studies with larger sample sizes. Moreover, gene–gene and gene–environment interactions should also be investigated in future studies.

## Supporting Information

S1 PRISMA Checklist PRISMA checklist. (DOC)
